# A 'good dyadic relationship' between older couples with one having mild cognitive impairment: a Q-methodology

**DOI:** 10.1186/s12877-022-03449-x

**Published:** 2022-09-21

**Authors:** Daphne Sze Ki Cheung, Grace Wing Ka Ho, Athena Chung Yin Chan, Ken Hok Man Ho, Robin Ka Ho Kwok, Yammie Pui Yan Law, Daniel Bressington

**Affiliations:** 1 School of Nursing, The Hong Kong Polytechnic University, Hong Kong SAR, China; 2 Department of Family Social Science, University of Minnesota, St Paul, USA; 3 The Nethersole School of Nursing, Faculty of Medicine, The Chinese University of Hong Kong, Hong Kong SAR, China; 4 St. James Settlement, Hong Kong SAR, China; 5 College of Nursing and Midwifery, Charles Darwin University, Casuarina, Australia

**Keywords:** Dyad, Caregiver, Relationship, Couplehood, Dementia, Q methodology

## Abstract

**Background:**

Cognitive impairment gradually brings changes to the relationship between older married couples. Therefore, this study aimed to understand the individual viewpoints of couple dyads on the important attributes of a 'good dyadic relationship' in the context of mild cognitive impairment (MCI), and to explore if the congruencies and discrepancies in their perceptions related to the quality and closeness of their relationship and well-being.

**Methods:**

Q-methodology was used to reveal the perceptions of a ‘good dyadic relationship’ among couples with one having MCI. The participating couples were separated in two rooms and independently ranked 18 relationship attributes from least to most important on a 7-point Q-sort response grid. All participants also completed a post-sort interview and surveys to assess their psychological well-being and closeness. Q-sorts were analyzed using by-person factor analysis.

**Results:**

Forty people with MCI and forty spousal partners completed the Q-sort. Three viewpoints, accounting for 48% of the total variance, were identified and were labeled ‘Provider,’ ‘Problem-solver,’ and ‘Partner.’ Different viewpoints of a ‘good dyadic relationship’ primarily varied by perceived importance of commitment, dedication, tolerance, and personal space. Despite these differences, there was wide consensus that respecting each other and cherishing the current moment are two universally salient attributes of a good relationship across all viewpoints. Couples with discrepant views scored significantly higher on perceptions of the quality of the relationship and closeness with the partner.

**Conclusions:**

This study advances the theoretical understanding of the dyadic relationship between couples with one having MCI, from both perspectives. MCI is a state in which couples can openly discuss their expectations. The findings provide practitioners with insights to work with couples experiencing MCI.

**Supplementary Information:**

The online version contains supplementary material available at 10.1186/s12877-022-03449-x.

## Background

Cognitive decline is expected as a part of physiological ageing. Mild cognitive impairment (MCI) refers to a state in which there is a noticeable accelerated decline in memory and executive functioning that is not severe enough to be considered dementia [1]. Although individuals with MCI may remain stable or improve in cognitive function, the changes in their memory or other cognitive domains often lead to strained relationships among family members [2]. There is ample evidence showing that cognitive impairment gradually alters the relationship between the person with cognitive impairment and family members, particularly the relationship between married older couples, due to a reduction in companionship and mutual support [3–5]. Likewise, the anticipatory grief related to relationship loss is significantly greater when experienced by the spouse than by the adult children of the person with cognitive impairment [6].

Coping with cognitive impairment is a dyadic affair [7]. The change in the marital relationship arising from MCI is attributable to multiple factors, for example, how a partner connects to the caregiver role, how a partner identifies himself/herself to the person with cognitive impairment, the current amount of effort required to maintain a relationship connection, and the dyad’s response to the illness [8]. Couples in which one partner has cognitive impairment often try to maintain a sense of ‘togetherness’ despite shifts in the balance of the relationship [5]. Hence, it is essential to identify the attributes of a good-quality dyadic relationship under the influence of cognitive impairment. This is important because spousal caregivers may associate relationship attributes such as a sense of duty, personal growth, acceptance and forgiveness, commitment to the relationship, and drawing strength from past challenges, with positive caregiving, relationship satisfaction, and well-being [9–11]. Therefore, understanding the dyad’s description of a ‘good dyadic relationship’ would help in the development of an intervention to preserve the quality of the relationship, which is a significant determinant of the well-being and quality of life of both the person with cognitive impairment and his/her spouse [9, 12].

Rippon et al. [13] highlighted the significance of considering the individual perspective of both partners. However, previous studies have mostly been from the perspective of the partner without cognitive impairment, and have unanimously assumed that the couple’s relationship is that of ‘caregiver-and-patient’ [14–16]. There has been increasing attention in research on the attributes of a quality dyadic relationship from the perspectives of both partners along the trajectory of cognitive impairment [17]. More importantly, labelling the couple dyad as being in a ‘carer-and-patient relationship’ violates the equity theory, which proposes that dyads strive to maintain a balance between the help that is given and the help that is received, and that an imbalance leads to distress for both members [18]. It is imperative to understand from both perspectives what constitutes good dyadic attributes under the influence of cognitive impairment.

Similarity of relationship standards and perceptual congruence can affect a couple’s marital satisfaction [19, 20]. The Interdependence Theory posits that values and goal incongruence can negatively affect the quality of a relationship [21]. However, studies have consistently reported incongruent perceptions between the person with cognitive impairment and that person’s family members, with regard to various aspects such as functional ability and coping [22]. It is unknown whether the congruency or discrepancy in perceptions of the attributes of a ‘good dyadic relationship’ is associated with satisfaction with the relationship among couples in which one partner has MCI. Therefore, the aim of this study was to understand individuals’ views of a ‘good dyadic relationship’ among older couples with one partner having MCI, and to explore whether congruent versus discrepant perceptions of a ‘good dyadic relationship’ within a couple is associated with individuals’ relationship satisfaction and well-being. The research questions are:

1. What are the prevailing views on the important attributes of a ‘good dyadic relationship’ in the context of MCI?
2. Do the relationship satisfaction, closeness, and individual well-being of couples with congruent views of what constitutes a ‘good dyadic relationship’ differ from those with discrepant views?

## Methods

### Study design

Q-methodology was employed to uncover prevailing views among older couples with one having MCI on the important attributes leading to a ‘good dyadic relationship’. This methodology includes a mix of quantitative and qualitative techniques designed to systematically examine people’s subjectivity through the operational medium of a Q-sort [23]. In the Q-sorting process, participants ascribe a psychological response to a set of stimulus items and provide a relative ranking of each item in comparison to other stimulus items. Thus, the completed Q-sort is considered a holistic and individualized construction of the participants’ views on a given topic that is wholly subjective, valid, and that cannot be deemed quantitatively superior or inferior to those of another [24]. Since exploring views of a ‘good dyadic relationship’ in the context of MCI is a highly subjective endeavor, and because participants might worry that expressing their expectations of a good relationship may give the impression that they are a bad or demanding spouse, Q-methodology is particularly suitable because it allows researchers to uncover and compare the prevailing viewpoints of participants about a phenomenon that could easily be affected by social desirability bias [25].

### Participants

A diverse and purposive sample of older couples with one partner having MCI was recruited. MCI is defined as cognitive decline greater than expected for an individual’s age and level of education [1]. People with MCI may present with memory impairment and/or deficit in other cognitive domains, leading to minor inconveniences in daily functioning, particularly in the instrumental activities of daily living, that are generally believed to be of insufficient severity to constitute a major disability [26]. The Clinical Dementia Rating Scale was used in the current study to screen for the severity of the cognitive impairment, with an overall score of 0.5 representing MCI [27]. The other inclusion criteria were that both partners of the couple should be: (a) living in the community, whether cohabitating or not; (b) aged 18 or above; (c) in a stable medical condition; (d) willing to join the study as a dyad; and (e) able to read and communicate in Chinese. Dyads were excluded if either partner had a critical psychiatric illness.

Q-methodological studies aim to identify and describe perspectives in depth [28]. A high volume of data prevents analyses of subtle patterns of meaning from being conducted [29]. Therefore, Q-studies typically involve between 40 and 60 purposively sampled participants with a maximum variation that would be considered more than adequate to elicit prevailing viewpoints [28]. In this study, a purposive sample of 40 couples with diverse backgrounds (i.e., in terms of the level of education, co-residence family structure, and socioeconomic status) was recruited through open advertisements in social media and referrals from participating elderly service centers in Hong Kong, with the service users being mainly Chinese.

### Procedures

#### A Q-methodology study consists of two phases

##### Phase 1: Creating a Q-sample

A Q-sample is a collection of items relevant to the topic that is used in subsequent Q-sorting [29]. The first phase focuses on exploring and understanding the discourse, or on communicable content related to a given topic [30]. The researchers developed the concourse of the Q-set by broadly looking into the literature regarding (a) the elements of a good couple relationship (which were usually derived from the relationships of undergraduates and young couples), (b) relationship maintenance among older adult couples, and (c) changes in the relationship of dyads having dementia. An electronic search of the literature in the PubMed, PsycINFO, and CINAHL databases was undertaken in March 2019, using the keywords [[couple relation*] OR [spous* relation*] OR [marital relation*] OR [intimate relation*] OR [dyad*]] AND [old OR elderly OR senior].

Fifteen reviews of qualitative/quantitative studies were reviewed (see the Supplementary Material 1) and 32 initial statements were proposed. These statements were translated into Chinese and reviewed by a panel of six experts (two academic experts on dementia care, one academic expert on mental health, two Q-methodology experts, and one sociologist), who subsequently refined the list to 18 statements through consensus.

The final statements and guides were pilot-tested with content experts, i.e., two older couples each with one partner having MCI, to promote the content and face validity of the Q-sample (see Table 1). In the pilot study, the participants reflected that some of the statements were not familiar to them; therefore, the research team edited the statements and provided a definition and example with reference to a dictionary to facilitate their understanding.

**Table 1:** The initial and final Q-sets

Initial Q-set	Final Q-set
Statements	
1. Commitment 2. Shared identity as a couple 3. Companionship 4. Face difficulties together 5. Interdependence 6. Appreciate one another 7. Sense of connection 8. Flexibility and openness 9. Respect one’s partner 10. Care 11. Empathy 12. Forgiveness and letting go 13. Strive to maintain the best interests of one’s partner 14. Maintain the dignity of one’s partner 15. Solve problems for one’s partner 16. Self-sacrifice 17. Hope 18. Create new perspectives 19. Acceptance 20. Humour 21. Selective comparison 22. Reminiscences 23. Live in the moment 24. Bring happiness to one’s partner 25. Patience and tolerance 26. Sense of security 27. Trust and interdependence 28. Intimacy 29. Open communication and interaction 30. Self-care 31. Perceived by others as a loving couple 32. Mind in synchronicity	1. Commitment and dedication 承擔 2. Companionship 陪伴 3. Appreciation 欣賞 4. Compromise 妥協 5. Respect 尊重 6. Empathy 感同身受 7. Self-sacrifice 無私付出 8. Hope 盼望 9. Acceptance 接納 10. Forgiveness 寬恕 11. Cherishing珍惜對方 12. Happiness 喜悅 13. Patience and tolerance 忍耐 14. Trust 信任 15. Intimacy 親密感 16. Openness 坦誠 17. Personal space 個人空間 18. Synchronicity 默契

##### Phase 2: Q-sorting

In this phase, the participants sorted the statements on a pre-defined continuum to construct a representation of their view [24]. The purpose of Q-sorting is to uncover what individuals view to be important attributes of a ‘good dyadic relationship’ among older couples with one partner having MCI. Therefore, participants completed the Q-sorting procedures independently (i.e. away from their partners) to elicit their views on what they perceive to be important. Couples were invited to complete the Q-sorts in separate rooms in a local community center providing elderly services. Participants were first given the stimulus question "What are the important attributes of a ‘good dyadic relationship’ in the context of MCI?" and sorted the Q-sample into two piles: ‘unimportant’ and ‘important’. Then, the participants were introduced to a 7-point Q-sort grid ranging from ‘least important, 1’ to ‘most important, 7’. The Q-sort grid consisted of 18 boxes, one for each statement, and was set up in a quasi-normal distribution; statements placed in the same column on the grid were assumed to share the same level of importance. Participants began by selecting from the two initial piles those statements that they felt were most important and most unimportant, and placed them on the extreme ends of the grid. Then, they were instructed to place the remaining statements across the grid based on their perception of their levels of importance relative to the others. They could move the statements around freely until they were satisfied with the complete sort. The participants were consulted on whether there were attributes they thought were important or unimportant related to the dyadic relationship that had not been listed in the Q-sample.

After submitting their finalized sorts, post-sort interviews were conducted where the participants were asked to elaborate on their overall view and impression, and on why they chose to place each of the four statements at two extreme ends of the grid. Overall, the participants were able to understand the instructions, and completed the sorting with minimal assistance.

Lastly, the participants completed a survey to provide information on their background, psychological well-being, and closeness in the dyadic relationship. Psychosocial well-being was measured with the Geriatric Depression Scale [31]. This scale consists of 15 items using a yes/no format, with higher scores indicating higher levels of depression. A high sensitivity (96%) and specificity (90%) on a cut-off score 7/8 were established. The participants were asked to rate their satisfaction with the dyadic relationship on a 10-point Likert scale, with higher scores indicating a better quality relationship. The Inclusion of the Other in the Self Scale [32] was used to measure the closeness of the dyadic relationship with good internal validity [33]. The participants were shown seven pairs of circles that ranged from just touching to almost completely overlapping. One circle in each pair was labelled ‘self’, and the second circle was labeled ‘partner’. They were then asked to select the one pair that best described their dyadic relationship. No overlap was scored as 1, while almost overlapping was scored as 7.

### Data analysis

To address research question #1, a by-person factor analysis was conducted using PQ Method 2.35 [34] to generate groups of participants who expressed similar views on their Q-sorts. This included the use of principal component analysis to first extract factors (i.e., groups of participants) with similar sorts based on total sort correlations; factors were considered significant when defined by at least two sorts and had an eigenvalue of greater than one, and varimax and manual rotation were used to enhance factor interpretability [29]. There is no gold standard to identify the best factor solution in Q-studies, therefore, different vantage points were adopted to identify the best solution for describing the different viewpoints. Initial assessments of iterations of 2-, 3-, and 4-factor solutions suggested that the participants’ views on ‘good dyadic relationship’ attributions were largely similar. The 3-factor solution was employed because it captured the fine distinctions between the different views.

Q-sorts defining each factor were weighted based on their factor loading to create a composite Q-sort, which is a summary Q-sort that uniquely represents the overall view of all participants defining that factor. These composite Q-sorts represent holistic constellations of views on the attributes of a ‘good dyadic relationship’ and were interpreted and assigned meaning by abductive reasoning [28, 35]. To interpret the composite Q-sorts, i.e., the rankings of statements on composite Q-sorts were compared and contrasted systematically to assess similarities and differences across views [29]. Similarities across views were identified to capture the general consensus on what participants generally perceive to be important or unimportant attributes of a ‘good dyadic relationship.’ Last, a narrative was generated for each composite Q-sort to provide a consolidated description of each viewpoint and the characteristics of the participants who defined them.

Furthermore, to address research question #2, the characteristics of individuals within couples with congruent versus discrepant views (i.e., defining the same or different factors) were compared using the Kruskal–Wallis test or Chi-squared test, analyzed with the SPSS 26 [36].

### Ethical considerations

Ethical approval was obtained from the University (HSEARS20190708002). The study was performed in accordance with the Declaration of Helsinki. An information sheet was given to all participants. After the purpose and procedures of the research were explained to them, their verbal and written informed consent to take part in the study was obtained. All analyses were anonymized, and the names of participants who gave quotes explaining their choice were replaced with an identifier. Each couple received an HKD$200 (approximately USD$25) supermarket coupon to compensate them for their time and travelling costs.

## Results

The study was conducted from December 2020 to June 2021. Forty-one couples were recruited, and 40 people with MCI and 40 partners completed the Q-sort. The only one person with MCI and one spousal partner did not complete the sort because they did not want to continue. The mean age of the participants was 75.5 (S.D. = 6.2). Most of them had received a primary education (i.e., the first stage of formal education, 45.1%), were retired or did not work (98.8%), and the mean duration of their marriage was 48.4 years (S.D. = 4.4).

The prevailing views on the important attributes of a ‘good dyadic relationship’ in the context of MCI were uncovered by the by-person factor analysis. Three factors resulted: Factor 1 Provider, Factor 2 –Problem-solver, and Factor 3 –Partner. The emergent factors comprised 60 defining sorts and accounted for 48% of the total variance (Factor 1: 23%; Factor 2: 13%; Factor 3: 12%), selected based on interpretability and eigenvalues (Factor 1: 25.72; Factor 2: 6.49; Factor 3: 6.18). The extent to which a participant’s individual Q-sort overlapped with each emerging factor was represented by the factor loading, ranging from between zero (no match) and one (perfect match) (see Supplementary Material 2). The profiles of all of the participants and the endorsers of each factor are listed in Table 2.

**Table 2:** Demographic characteristics

	**Full sample** (*N* = 82)		**Factor 1**(*n* = 33)		**Factor 2** (*n* = 13)		**Factor 3**(*n* = 14)		*p*
	N	%	n	%	n	%	n	%	
**Role**									
Person with MCI	41	50.0	19	57.6	5	38.5	6	42.9	.478^a^
Spouse	41	50.0	14	42.4	8	61.5	8	57.1	
**Age**									
Mean (SD)	75.5 (6.2)		76.2 (5.1)		73.5 (6.4)		75.1 (9.1)		.320^b^
**Gender**									
Male	41	50.0	21	63.6	3	23.1	9	64.3	**.033**^**a**^
Female	41	50.0	12	36.4	10	76.9	5	35.7	
**Education level**									
Nil / pre-primary	14	17.1	4	12.1	2	15.4	3	21.4	.736
Primary	37	45.1	15	45.5	8	61.5	6	42.9	
Secondary	24	29.3	9	27.3	3	23.1	4	28.7	
University or above	7	8.5	5	15.2	0	0.0	1	7.1	
**Years of Education**									
Mean (SD)	8.2 (4.4)		9.21 (4.9)		7.85 (3.5)		7.00 (4.0)		.331^b^
**Marital Status**									
Married	82	100.0	33	100.0	13	100.0	14	100.0	N.A
Other	0	0.0	0	0.0	0	0.0	0	0.0	
**Years of Marriage**									
Mean (SD)	48.4 (6.8)		48.24 (5.4)		47.31 (9.8)		48.29 (5.4)		.343^b^
**Religious Beliefs**									
None	52	63.4	21	63.6	9	69.2	9	64.3	.994^a^
Christianity	10	24.4	7	21.2	2	15.4	3	21.4	
Buddhism	10	12.2	5	15.2	2	15.4	2	14.3	
Other	0	0.0	0	0.0	0	0.0	0	0.0	
**Living Arrangement**									
With spouse only	54	65.9	21	63.6	11	84.6	8	57.1	.274^a^
Spouse & others	28	34.1	12	36.4	2	15.4	6	42.9	
**Number of Household Members**^**c**^									
Mean (SD)	1.7 (1.1)		1.64 (1.1)		1.31 (0.9)		1.93 (1.4)		.262^b^
**Employment Status**									
Retired	81	98.8	32	97.0	13	100.0	14	100.0	.660^a^
Full-time	0	0.0	0	0.0	0	0.0	0	0.0	
Part-time	1	1.2	1	3.0	0	0.0	0	0.0	
Temporary	0	0.0	0	0.0	0	0.0	0	0.0	
**Perceived Economic Status**									
Very sufficient	6	7.3	3	9.1	0	0.0	1	7.1	.689^a^
Sufficient	59	72.0	24	72.7	11	84.6	9	64.3	
Insufficient	17	20.7	6	18.2	2	15.4	4	28.6	
Very insufficient	0	0.0	0	0.0	0	0.0	0	0.0	
**Lawton’s Instrumental Activities of Daily Living total score (Range: 0 – 27)**^**d**^									
Mean (SD)	23.8 (4.5)		24.7 (3.9)		24.4 (3.8)		22.8 (5.0)		.454^b^
**Geriatric Depression Scale (Range: 0 – 15)**									
No depressive symptoms	72	87.8	31	93.9	10	76.9	12	85.7	.254^a^
Significant depressive symptoms	10	12.2	2	6.1	3	23.1	2	14.3	
Mean (SD)	3.3 (3.7)		2.42 (2.8)		4.23 (4.2)		4.14 (4.3)		.303^b^
**Perceived Quality of Relationship (Range: 0 – 10)**									
Mean (SD)	7.9 (1.7)		7.88 (1.6)		7.08 (2.4)		8.46 (1.2)		.156^b^
**Inclusion of the Other in the Self Scale**									
No overlap	1	1.2	0	0.0	1	7.7	0	0.0	.105^a^
Little overlap	3	3.7	3	9.1	0	0.0	0	0.0	
Some overlap	5	6.1	2	6.1	1	7.7	0	0.0	
Equal overlap	6	7.3	2	6.1	2	15.4	0	0.0	
Strong overlap	10	12.2	5	15.2	0	0.0	2	14.3	
Very strong overlap	25	30.5	9	27.3	7	53.8	3	21.4	
Most overlap	31	37.8	12	36.4	2	15.4	9	64.3	
Missing	1	1.2	0	0.0	0	0.0	0	0.0	
Mean (SD)	5.7 (1.5)		5.6 (1.6)		5.23 (1.7)		6.5 (0.8)		**.047**^**b**^

^a^ Chi-Square Test

^b^ Kruskal–Wallis Test;

^c^ excluding the person with MCI;

^d^for the sample with MCI only

### Similarities across factors

Correlations between the three factors ranged from 0.38 to 0.51, indicating that there were considerable similarities across viewpoints. In addition, participants from all three factors generally agreed on the most important attributes of a ‘good dyadic relationship’ in the context of MCI. These were (a) respect for each other and (b) cherishing the current moment. These thoughts are reflected in the following comments from post-sort interviews:

*‘All the attributes listed are important in a relationship. However, mutual respect is the foundation of everything in a relationship. I respect her and she respects me, as we have always in the past fifty years.’* (Case 2, the person with MCI, a 79-year-old male)

*‘I don’t know how long we shall live and perhaps one day one of us will die unexpectedly. We are old and won’t live long. Therefore, I cherish the moments that she [the partner with MCI] is with me.’* (Case 41, partner, a 69-year-old male)

### Differences across factors

Despite general similarities found across the three factors, some differences in what people with MCI and their partners identified as important attributes in a ‘good dyadic relationship’ in the context of MCI were observed. The distinctive features of the three factors are described below and in Table 3.

**Table 3:**
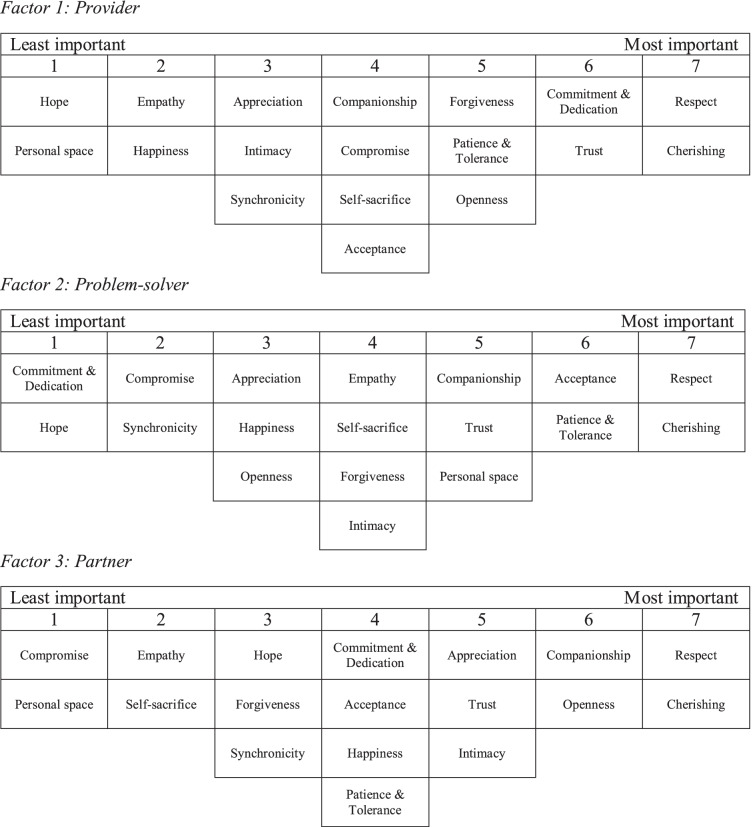
Statement rankings for composite Q-sorts across the three factors

#### Factor 1: provider

This factor was endorsed by 33 participants, 19 of whom had MCI and 14 of whom were a spouse. The majority of the participants were male (63.6%) and living in rented public housing (42.5%). Descriptive comparisons suggest that these participants had the longest mean number of years of education (9.21 ± 4.92 years) and the lowest Geriatric Depressive Scale scores (2.42 ± 2.78) compared with the other two groups, although the differences did not reach the level of statistical significance.

Those endorsing this view placed an emphasis on commitment and dedication as important attributes of a ‘good dyadic relationship’. *‘The more you want, more likely you will be disappointed [because of his cognitive impairment]. Thus, I do more than I would expect in return’* (Case 25, the spouse, a 79-year-old female). A participant with MCI reflected that she and her spouse had contributed a lot to the family when they were younger. Her husband went to work, and she raised three children at home. *‘I’m frail and weak now. I rely on him, and he is committed to caring for me too’* (Case 23, the person with MCI, a 68-year-old female).

The participants appeared to feel that there was an obligation to contribute in a relationship. A spouse said that his wife had cognitive decline, and he needed to take care of a lot of trivial matters at home, but that he was fine with that because *‘A relationship can only be maintained through commitment and being conscious of one’s partner’s feelings’* (Case 10, the spouse, a 79-year-old male). On the other hand, they were neutral about the idea of the importance of companionship and perceived personal space as the least important attribute. *‘He always needs me to stay with him. I cannot leave him alone. Not to mention that we live together in a small apartment’* (Case 12, the spouse, an 80-year-old female).

#### Factor 2: problem-solver

Thirteen participants (5 MCIs and 8 partners) endorsed this viewpoint. Significantly different from the other two factors, the majority of them were female (76.9%). Among the factors, this factor contained the largest portion of partners living with only the spouse in the same household (84.6%) and living in privately-owned housing (53.8%), as well as the largest proportion of those who perceived that their economic means was sufficient (84.6%), although the difference did not reach the level of statistical significance. Similarly, the participants in Factor 2 were younger than those of the other factors, had more severe geriatric depressive symptoms, and had the poorest rating of the quality of their dyadic relationship. There was the least amount of overlap with their partner, indicating the less closeness of their relationship, which was statistically significant from the other two factors.

Endorsers of Factor 2 ranked acceptance, patience, and tolerance as being of higher importance in a relationship as compared to Factor 1 and 3, and commitment and hope as being the least important. This suggests that they were focused on the problems they are currently facing, and on the importance of demonstrating acceptance and tolerance. A spouse said, *‘My partner cannot be changed. The only thing I can do is to accept and bear with it [the difficulty]’* (Case 12, the spouse, a 72-year-old female). Another spouse shared similar views, but she highlighted individuality in the relationship and said, *‘We are two individuals and may have different thoughts. I cannot force my partner to be the same as I am…. So I bear with it [the temper of the partner]’* (Case 37, the spouse, an 84-year-old female). At the same time, they rated the attribute of synchronicity as the lowest among the three factors. Not only the partner, but also the person with MCI had the same idea, and said, *‘Even though we are partners, I cannot lose myself [my identity] for him’* (Case 43, the person with MCI, a 66-year-old female). They seldom think of the future and their hopes, but focus on the current situation *‘We have lived long enough. I have no unfulfilled wishes. I seldom think of the future. There is too much to take care of now’* (Case 21, the person with MCI, a 71-year-old male).

#### Factor 3: partner

This factor was endorsed by 14 participants (6 people with MCI and 8 spouses). As seen from the descriptive statistics, the majority were male (64.3%) and this group had the fewest number of years of formal education compared with those in the other factors. They also had the highest rating in both the perceived quality of the relationship and the level of closeness in the relationship, compared with endorsers of Factors 1 and 2. Yet their depressive symptoms were as high as those of Factor 2.

Similar to those endorsing Factor 1, endorsers of Factor 3 regarded personal space as unimportant, but valued companionship, intimacy, and openness in a relationship. Unlike those who endorsed Factors 1 and 2, who focused on what they could contribute in a relationship, a spouse in Factor 3 said, ‘*My partner is frail. I want to stay with my partner as much as I can*’ (Case 20, the spouse, a 76-year-old female). Another participant said, *‘We always stick together and hold hands when we go out. Our neighbours always kidding us, saying that whenever they see me, they can see my partner’* (Case 22, the person with MCI, an 85-year-old male). Those in Factor 3 also appraised their relationship positively by affirming the importance of appreciation, which was not the case with those in Factors 1 and 2. *‘My partner is very stubborn, but I appreciate her contribution to the family. I value her so much’* (Case 12, the person with MCI, a 77-year-old male). As with those in Factor 1, they rated personal space as the least important attribute. Participants in Factor 1 gave this a low rating because they might think they were obligated to stay with their partner and provide assistance and were committed to doing so, as reflected from the quote of Case 12. Yet the participants in Factor 3 interpreted the matter differently because they trusted their partner. One stated, *‘I have no secrets from my partner. We understand each other well. I tell him everything. I don’t need personal space’* (Case 22, the person with MCI, a 65-year-old female). This is consistent with those in Factor 3 having the highest score among the three factors in perceived closeness as measured with the Inclusion of the Other in the Self score, although statistical significance was not reached, probably because of the small sample.

### Dyads with congruent views and discrete views

Forty-two participants were in a couple where both partners provided a defining sort. Among them, eleven dyads endorsed the same factor (i.e., congruent views), while ten dyads endorsed different factors (i.e., discrepant views). Among individuals who were part of a couple with discrepant views, perceptions of the quality of the relationship (*p* = 0.005) and closeness with the partner (*p* = 0.023) were significantly better than among couples with congruent views (see Table 4 for details).

**Table 4:** Comparison of the perceived quality of the relationship, well-being, and closeness with one’s partner, between dyads with congruent views and discrete views

	With congruent views (*n* = 11 dyads)	With discrete views (*n* = 10 dyads)	*p^*
	Mean (SD)	Mean (SD)	
Perceived quality of the relationship (range: 1 – 10)	7.13 (1.3)	8.1 (1.5)	**.005**
Geriatric Depression Scale (range: 0 – 15)	3.0 (2.9)	4 (4.2)	.360
Inclusion of the Other in the Self Scale (range: 1 – 7)	5.3 (1.1)	6.1 (0.9)	**.023**

Note: ^ Kruskal–Wallis test

## Discussion

The spousal relationship has consistently been reported as having changed since one member of the dyad developed cognitive impairment. This study gave voice to individuals and their partners who are affected by MCI by uncovering their views on the important attributes of a ‘good dyadic relationship’. Three new findings were made in this study. First, there was a consensus on the importance of respecting each other and cherishing the current moment in a dyadic relationship when anticipating cognitive impairment. Second, nuances were uncovered in relation to the specific attributes necessary to achieve a ‘good dyadic relationship’, which resulted in three varying views: Provider, Problem-solver, and Partner. Third, couples with discrepant views reported having a better-quality relationship and greater closeness than those with congruent views.

Although some people with MCI function fairly well, a systematic review found that those with MCI felt that they were less relied upon and were a burden to others, and were frustrated with the reactions of family and friends to their reduced cognitive abilities [37]. They might have felt that they were no longer on ‘equal terrain’ with each other, and there was an experiential shift from the norm of their pre-existing relationship roles, which contributed to tension in the relationship [38]. These earlier studies suggest that the couple relationship had changed into that of a ‘patient-caregiver’ relationship. Perhaps for that reason, the participants in this study treasured the respect of each other to alleviate tension in the relationship. The finding is consistent with that of other studies investigating factors related to satisfaction in the marital relationship. For example, in satisfactory relationships, couples were respecting and listening to the choices made by their partner [39]. Even when disputes and difficulties arose, the open communication and respect between family members made it possible to find a solution out of concern for each other and in view of the others’ distress [40].

People with MCI and their partners emphasized cherishing the current moment, possibly because of the nature of MCI. People with MCI and their partners reported that their memory problems were not ‘extremely debilitating’, but that they were sometimes confused by changes that they observed, which could be unpredictable and transient [38]. Wadham et al. further explained that couples might perceive the future as uncertain but the knowledge of inevitable deterioration provoked distressing emotions, including a sense of hopelessness, powerlessness, and futility [5]. MCI may prompt a re-evaluation of their life together, helping them to appreciate what they had previously taken for granted, and to cherish the present moment in order to cope with negative emotions. Mindfulness-based interventions have been widely used among partners or significant others of those with cognitive impairment in order to promote psychological wellness [41, 42]. These are grounded on the philosophy of being non-judgemental and focusing on the present moment, preventing one from thinking negatively about the future. Yet implementing a mindfulness-based intervention using a dyadic approach is a new perspective that deserves more attention [43].

Furthermore, in the process of findings ways to support a ‘good dyadic relationship’, this study identified three types of identity presented by people with MCI and their partners. We named these the 3Ps: Provider, Problem-solver, or Partner. Of note, In Factor 1 (Provider), there were spouses and persons with MCI. It is not difficult to regard a partner as a care provider, yet finding the person with MCI acting as a provider in a relationship was unanticipated. In a healthy and mutually beneficial partnership, reciprocity is a basic principle of social exchange as it addresses the issue of equality through ‘give’ and ‘take’ [44]. However, the majority of studies examining MCI labeled the couple dyad as being in a ‘patient-caregiver relationship’, which violates the principle of equity. Failed reciprocity between dyads might be associated with poorer well-being in the person with a disability and his/her partner [45]. People with MCI are not totally incapable and can also be serving as a provider in a relationship. Through providing support (especially emotional support), the well-being of the providers themselves would be enhanced.

MCI is a family issue that may affect both partners. Therefore, we uncovered the problem-solver as a unique identity that was deemed to be important for a ‘good dyadic relationship’. This was because those participants considered patience, acceptance, and tolerance as key to facing their present challenges. Those participants further rated commitment as the least important of the attributes. People with MCI may perceive as a threat the loss of their own identity as a problem-solver because of the unpredictable progression of MCI; the resulting feeling of diminished significance in the relationship may lead to depression [46]. For the partner without cognitive impairment, the Systemic-Transactional Model can explain their views [47]. Such partners supported the other partner by acting as a problem-solver, but if they were unwilling or showed that their support should not be necessary, this was referred to as ambivalent dyadic coping. Regarded as a negative form of dyadic coping, studies reported that ambivalent dyadic coping was associated with poorer relationship satisfaction and depression, which warrants our attention [48, 49]. Our findings were consistent with the existing knowledge that participants in Factor 2 were found to have a lower quality of relationship and less closeness with their partner.

The Factor 3 endorsers regarded companionship and openness as important attributes of a ‘good dyadic relationship’, which is consistent with the findings of a recent interpretative phenomenological study that examined how relationships are described by the spouse of a person with cognitive impairment [50]. In that study, spouses regarded themselves as distinct individuals but shared almost everything in their lives with the person with cognitive impairment by developing a partnership of doing things together. This is coherent with the ‘Factor 3: Partner’ of our findings, but we built upon this knowledge by showing that a person with MCI could have similar perspectives. However, as maintaining a sense of partnership within a relationship is encouraged in couple therapy, this group of participants reported greater depressive symptoms warrants attention. It echoes our previous study showing that a more interdependent premorbid relationship may lead to a stronger sense of grief [6].

“Birds of a feather flock together” and “opposites attract” are two contrasting statements about intimate relationships that have been debated in the past half-century without a firm conclusion [51]. We found that couples with discrepant views of what constitutes a ‘good dyadic relationship’ (i.e., in different Factors) reported better perceptions of the quality of their dyadic relationship and greater relationship closeness than those with coherent perspectives. There are a handful of research studies investigating if the similarity in personality, political views, coping style, demo sociographic background, and gender affect spousal relationship satisfaction [51–54]. Future research may explore how the discrete views of relationship attributes within couples, contribute to relationship satisfaction or closeness through qualitative inquiry, and may further inform couple-based intervention development.

MCI is a risk factor for developing dementia. Sustaining a close emotional relationship between partners is crucial to the persons with dementia and maintaining their sense of personhood [55]. Identifying the attributes of a ‘good dyadic relationship’ as early as in the MCI stage may be helpful to increase the potential for supporting good quality and close spousal relationships, thus contributing to a sustained sense of personhood in dementia.

### Limitations

Despite carrying out purposive sampling in the attempt to maximize variations based on demographic background, it is possible that we may have missed couples with other unique viewpoints. In addition, this study examined relationships in the context of Chinese culture, so it may not be representative of the situation in other cultural contexts, particularly since perceptions related to marital relationships are influenced by cultural beliefs [56]. Therefore, we suggest extending the research to the older population, like those without cognitive impairment, and with other cultural backgrounds. We compared the well-being, relationship quality, and relationship closeness of those with congruent and discrepant views, but the results need to be interpreted with caution due to the small sample size. The findings are meant to generate further inquiries, which can be addressed in future research.

## Conclusions

This study investigated views of important attributes of ‘good dyadic relationship’ by involving both individuals in the spousal relationship. The results informed us that mutual respect and cherishing the current moment are the two universally important attributes in a dyadic relationship in the context of MCI. Three types of identity were revealed in a relationship: provider, problem-solver, and partner. The findings remind us that people with MCI and their partners are not always homogenous. Their roles and expectations of the relationship should be evaluated before any therapeutic interventions are carried out.

## Supplementary Information

**Additional file 1.**

## Data Availability

The datasets generated and/or analyzed during this current study are not publicly available due to ongoing use of data set but are available from the corresponding author on reasonable request.
