# Supplementary material for: A 'good dyadic relationship' between older couples with one having mild cognitive impairment: a Q-methodology
Source: BMC Geriatr. 2022 Sep 21;22:764. doi: 10.1186/s12877-022-03449-x (PMC9494827; doi:10.1186/s12877-022-03449-x)
Supplement: Supplementary file 1 — Additional file 1. [file 12877_2022_3449_MOESM1_ESM.docx]

**Supplementary Material 1:**

*Fifteen reviews for creating Q statements*

Factors contributing to relationship and its indicator (change)

1. Hennings J, Froggatt K. The experiences of family caregivers of people with advanced dementia living in nursing homes, with a specific focus on spouses: A narrative literature review. Dementia. 2019;18(1):303-22. <https://doi.org/10.1177/1471301216671418>
2. Hong SC, Coogle CL. Spousal Caregiving for Partners With Dementia: A Deductive Literature Review Testing Calasanti’s Gendered View of Care Work. J Appl Gerontol. 2016;35(7):759-87. <https://doi.org/10.1177/0733464814542246>
3. Evans D, Lee E. Impact of dementia on marriage: A qualitative systematic review. Dementia. 2014;13(3):330-49. <https://doi.org/10.1177/1471301212473882>
4. Karantzas GC, Romano D, Lee J. Attachment and aged care: a systematic review of current research. Curr Opin Psychol. 2019;25:37-46. <https://doi.org/10.1016/j.copsyc.2018.02.016>
5. Nelis SM, Clare L, Whitaker CJ. Attachment in people with dementia and their caregivers: A systematic review. Dementia. 2014;13(6):747-67. <https://doi.org/10.1177/1471301213485232>
6. Eriksen S, Helvik AS, Juvet LK, Skovdahl K, Førsund LH, Grov EK. The Experience of Relations in Persons with Dementia: A Systematic Meta-Synthesis. Dement Geriatr Cogn Disord. 2016;42(5-6):342-68. <https://doi.org/10.1159/000452404>
7. Conway ER, Watson B, Tatangelo G, McCabe M. Is it all bleak? A systematic review of factors contributing to relationship change in dementia. Int Psychogeriatr. 2018;30(11):1619-37. <https://doi.org/10.1017/s1041610218000303>
8. Ablitt A, Jones GV, Muers J. Living with dementia: a systematic review of the influence of relationship factors. Aging Ment Health. 2009;13(4):497-511. <https://doi.org/10.1080/13607860902774436>
9. Safavi R, Berry K, Wearden A. Expressed emotion in relatives of persons with dementia: a systematic review and meta-analysis. Aging Ment Health. 2017;21(2):113-24. <https://doi.org/10.1080/13607863.2015.1111863>
10. Lloyd J, Patterson T, Muers J. The positive aspects of caregiving in dementia: A critical review of the qualitative literature. 2016;15(6):1534-61. <https://doi.org/10.1177/1471301214564792>

Impact by developmental stage

1. Holdsworth K, McCabe M. The impact of younger-onset dementia on relationships, intimacy, and sexuality in midlife couples: a systematic review. Int Psychogeriatr. 2018;30(1):15-29. <https://doi.org/10.1017/S1041610217001806>
2. Holdsworth K, McCabe M. The impact of dementia on relationships, intimacy, and sexuality in later life couples: An integrative qualitative analysis of existing literature. Clin Gerontol. 2018;41(1):3-19. <https://doi.org/10.1080/07317115.2017.1380102>

How does relationship affect different outcomes?

1. Edwards HB, Ijas S, Whiting PF, Leach V, Richards A, Cullum SJ, et al. Quality of family relationships and outcomes of dementia: a systematic review. BMJ Open. 2018;8(1):e015538. <https://doi.org/10.1136/bmjopen-2016-015538>
2. Quinn C, Clare L, Woods B. The impact of the quality of relationship on the experiences and wellbeing of caregivers of people with dementia: A systematic review. Aging Ment Health. 2009;13(2):143-54. <https://doi.org/10.1080/13607860802459799>

Resilience

1. Teahan Á, Lafferty A, McAuliffe E, Phelan A, O'Sullivan L, O’Shea D, et al. Resilience in family caregiving for people with dementia: A systematic review. Int J Geriatr Psychiatry. 2018;33(12):1582-95. <https://doi.org/10.1002/gps.4972>

**Supplementary Material 2**

**Table 1.** Factor matrix of the loadings of all participants on each factor

| Participant | Factor 1 | Factor 2 | Factor 3 |
| --- | --- | --- | --- |
| M1 | .1507 | .0115 | .4705* |
| S1 | .3453 | -.0478 | .5539* |
| M2 | .8128* | -.0532 | .0647 |
| S2 | .5132* | .0339 | .3663 |
| M3 | .3935 | .4652 | .4280 |
| S3 | .5603 | .6019^ | -.2860 |
| M4 | .0094 | .7766* | -.1681 |
| S4 | .1647 | .6150* | .2560 |
| M5 | .5335* | .232 | .3574 |
| S5 | .7048* | .3910 | -.0038 |
| M6 | .7345* | .0103 | .3833 |
| S6 | .3565 | .2988 | -.3168 |
| M7 | .3285 | .4414 | .3658 |
| S7 | .2748 | .5466 | .5290 |
| M8 | -.0691 | .1588 | .2713 |
| S8 | .6362* | .1978 | .2688 |
| M9 | .3021 | -.4599 | .1137 |
| S9 | .1464 | .6584* | .0837 |
| M10 | .3300 | -.0209 | .6001* |
| S10 | .3308 | .4994* | -.0646 |
| M11 | Did not complete the sort | | |
| S11 | .7075* | .0972 | .2349 |
| M12 | .2817 | .0168 | .7244* |
| S12 | .5543* | .2473 | .2925 |
| M13 | -.0142 | .3143 | .3780 |
| S13 | -.1340 | .1711 | .5059* |
| M14 | .7018* | .1763 | .2505 |
| S14 | .3931 | .2034 | .3368 |
| M15 | -.0203 | .6036* | -.1216 |
| S15 | Did not complete the sort | | |
| M16 | .5855* | .3696 | .0757 |
| S16 | .5391* | .4623 | .1329 |
| M17 | .1902 | .7546* | -.0776 |
| S17 | .0536 | .4085 | .4357 |
| M18 | .2496 | -.1920 | .4141 |
| S18 | .1984 | .1709 | .5403* |
| M19 | .3714 | .4715* | .1113 |
| S19 | .2918 | .2711 | .1542 |
| M20 | .5203 | .2885 | .6951* |
| S20 | .6136* | .2389 | -.0033 |
| M21 | .7725* | -.1211 | .0385 |
| S21 | .7282* | .0802 | .0848 |
| M22 | .1468 | .3724 | .4664* |
| S22 | .5616* | .4540 | -.0919 |
| M23 | .6284* | .1233 | .1988 |
| S23 | .3947 | .3712 | .1725 |
| M24 | .6924* | -.1089 | .3594 |
| S24 | .0503 | .2437 | .2611 |
| M25 | -.0565 | -.2141 | .4770* |
| S25 | .6611* | .0753 | .4138 |
| M26 | .5702* | .1060 | .2961 |
| S26 | .6429* | .3410 | .2643 |
| M27 | .1479 | .3359 | .2783 |
| S27 | .4238 | .0720 | .1746 |
| M28 | .7922* | .2314 | -.2507 |
| S28 | .6632* | -.0344 | .0683 |
| M29 | .8137* | .1381 | .0635 |
| S29 | .5186 | .5924* | .1747 |
| M30 | .7246* | .0397 | .1587 |
| S30 | .6068* | .0810 | -.0353 |
| M31 | .7347* | .2820 | .1441 |
| S31 | .4542 | .1744 | .2399 |
| M32 | .1475 | -.0557 | .4619^ |
| S32 | -.0068 | .7923* | .2120 |
| M33 | .5621* | .1653 | .4923 |
| S33 | .2342 | .3094 | -.0359 |
| M34 | .7036* | .0637 | .2611 |
| S34 | .4317 | .4122 | .5629 |
| M35 | .7867* | .2755 | .2393 |
| S35 | .0023 | .7376* | .4026 |
| M36 | .7336* | -.0573 | .2001 |
| S36 | .2190 | .3345 | .6057* |
| M37 | .5284* | .2324 | .3534 |
| S37 | .5345 | .5045 | -.3118 |
| M38 | .6066* | .3994 | .1941 |
| S38 | .7350* | .2001 | .2191 |
| M39 | .3664 | .2986 | .1395 |
| S39 | .1093 | .5045* | .3538 |
| M40 | .1949 | .2661 | .5971* |
| S40 | .3414 | .3814 | .7539* |
| M41 | .1406 | .5912* | .3263 |
| S41 | -.0878 | -.1124 | .6531* |
| % Variance | 23 | 13 | 12 |
| *Significant loading determined by automatic flagging; ^loading determined manually | | | |
